# Medicine in Arabia
***Arabian Medicine.** The Fitzpatrick Lectures delivered at the Royal College of Physicians 1919-20. By Edward G. Browne, M.B., F.R.C.P., Adams Professor of Arabic, Cambridge. (Cambridge: University Press. 1921. Demy 8vo. Pp. 138. Price 12s. net.)


**Published:** 1921-11

**Authors:** 


### THE REVIEW OF THE MONTH.

### MEDICINE IN ARABIA.*

It has ever been the proud boast of the medical profession that it can claim amongst its members men who have achieved greatness in other branches of knowledge than in that of medicine in the strict sense of the term. Lord Bacon, in his "Advancement of Learning," perhaps somewhat cynically, attributes this interest in matters not immediately connected with their profession to the failure of the public to distinguish between skill and quackery, between knowledge and effrontery, and to the unequal rewards which accrue. "Therefore it often falls out that the impostor beares away the Prize, virtue the Censure . . . and I cannot much blame Physitians, if they use commonly to intend some other Art or Practice which they fancy more than their Profession: for you shall have of them Poets, Antiquaries, Critiques, Politiques, Divines, etc., and in these artes better seen, than in their own profession." (Lib. iv., cap. 2, 1640 edition.) Be this as it may, the world is all the richer by this pursuit of the byways of knowledge. There are few subjects more fascinating than the history of the medical lore of the compatriots of Muhammad, for by them was kept burning the lamp of knowledge when the learning of Europe suffered the eclipse of the Dark Ages. When Alexandria fell the centre of gravity of intellectual activity shifted eastwards to the impetuous and quick-witted Arabs, at that time but recently emerged from a condition little removed from barbarism. These, under a succession of enlightened Caliphs, eagerly assimilated the philosophy and science of the disrupted Byzantine Empire. One of the conditions of peace imposed upon the Emperor Nicephorus by his conqueror Hárúnu'r Rashid was the delivery of MSS. of Greek masterpieces. At his command these were translated into Arabic, which was the learned language of the whole Arabic world. The history of this transmission of medical science is unfolded in Professor Browne's Fitzpatrick Lectures for 1919 and 1920. Those who took part in this work of transmission were largely Jews, Greeks, and Nestorian Christians from Armenia, and but rarely genuine Arabs. It was to the Nestorian Christians, who had been driven from Edessa, that the famous medical school of Jundi-shapur, in what is now known as the province of Khuzistan, in South-West Persia, owed its great development. This school was at the time of the birth of the Prophet at the height of its glory, and it was there that Hippocrates and Galen were first translated into Syriac. Moreover, hither were brought also the works of Indian medical writers, so that it thus became the meeting-place of Eastern and Western learning. A great hospital came into being, which flourished for 250 years under six successive generations of the Bukht-Yishu (i.e. "Jesus hath delivered") family. Another Christian physician of this school, Hunayn ibn Ishaq (i.e. "John, Son of Isaac") was a most prolific translator from the Greek into Arabic or Syrian, according to the needs of his Christian or Muslim readers. In these translations some of the original writings now lost have fortunately been preserved. But the most highly esteemed of medical writers who wrote in Arabic were Persians by race. They were largely independent of the Greek medical teaching, and compiled works based to a considerable extent upon personal observation and experience. It is this fact that adds interest to their writings, and marks out the period 850 to 1000 A.D. as one of remarkable importance in the history of medicine. Professor Browne selects for consideration four writers of special eminence—viz. Ali ibn Rabban, whose name betokens his Jewish origin; Rází (Rhazes) Ali ibnu'l Abbas (Haly-Abbas) and Ali Husayn ibn Síná (Avicenna), each of whom was a prolific writer of medical treatises. The first-mentioned produced a compendium of science, compiled not only from Hippocrates, Aristotle, and Galen, but also from Arabic and Indian sources. To this he gave the title "The Paradise of Wisdom." It is, however, to his pupil Rází that we owe the first original contributions to the medicine of the period. As the first physician to give clear descriptions of smallpox and measles, he has rightly earned a place in the temple of fame. One of his own compatriots (c. 1240 A.D.) thus writes of him: "There are many accounts by ar-Rází as to what he achieved by his skill in the art of medicine, his unique attainments in the healing of the sick, his deduction of their condition through his skill in prognosis, and the information which he gave as to their treatment unto the like of which but few physicians have attained."

With this eulogium we are prepared to agree, for he built upon a sound foundation of clinical experience, and his writings greatly influenced mediæval medical opinion. But the most famous of all was the fourth of those mentioned above, best known by his Latinised name Avicenna, but entitled amongst his contemporaries and successors the "Chief Master" or the "Second Teacher" (Aristotle being considered the pre-eminent teacher of mankind). Born about 980 he early acquired a reputation as a philosopher, as a physician, and as a poet. It is interesting to find that some of the quatrains attributed to Omar Khayyam may be with greater accuracy attributed to Avicenna. His *magnum opus* is his encyclopædic Qánún, of which a twelfth-century Persian writer speaks thus: "Could Hippocrates and Galen return to life it would be proper that they should do reverence to this book. . . . Whoever, therefore, finds fault with these two great men (i.e. Aristotle and Avicenna) will have cast himself forth from the

* **Arabian Medicine.** The Fitzpatrick Lectures delivered at the Royal College of Physicians 1919-20. By EDWARD G. BROWNE, M.B., F.R.C.P., Adams Professor of Arabic, Cambridge. (Cambridge: University Press. 1921. Demy 8vo. Pp. 138. Price 12s. net.)

**The Review of the Month—(*cont.*).**

fellowship of the wise, ranked himself with mad men, and revealed himself as fit company only for fools. May God by His Grace and favour keep us from such stumblings and vain imaginings." For a time the Qánún, translated into Latin, overshadowed even the fame of Galen, and for four centuries formed the chief text-book of European medicine. In a people possessed of such intellectual activity it is natural that the social position of the physician was a distinguished one, and that he was amply rewarded by those who experienced his skill. Anecdotes of the exhibitions of this wonderful skill became popular. Professor Browne gives a number of these relating to the more famous doctors. Probably the best known of these is the story of two rival physicians who agreed to a professional duel. The first prepared a poisonous draught of terrible potency which his opponent drank, but promptly neutralised by an antidote of equal power. It was now his turn, so, breathing a spell over a rose he offered it to his rival, who put it to his nostrils only to "Die of a rose in aromatic pain!"

Such is the power of suggestion, which was in truth the basis of many of the curative measures of the time, for Mysticism and Occultism has ever had a powerful attraction for the Eastern mind. This attraction is further seen in the belief in the supposed influence of certain numbers, which, as the foundation of astrology, exercised so great a power in Europe in later times, and even to-day holds a strong sway in popular thought.

Such, in brief outline, are some of the points of interest in Professor Browne's fascinating book.

